# Indigestion

**Published:** 1901-02

**Authors:** C. F. Ulrich

**Affiliations:** Wheeling, West Virginia; Ex-President Board of Education; Ex-President National Medical Society and Medical Society of Wheeling; President German Pioneer Society; Surgeon U. S. Marine Hospital Service; Member Board of Water Commissioners


For Texas Medical Journal.

### Indigestion.

BY C. F. ULRICH, A. M., M. D., WHEELING, WEST VIRGINIA.
Ex-President Board of Education; Ex-President National Medical Society and Medical Society of Wheeling; President German Pioneer Society; Surgeon U. S. Marine Hospital Service; Member Board of Water Commissioners.

Dyspepsia has been for generations the prevailing disease and, for lack of care of the digestive apparatus, the besetting sin of the American people. Even the pictoral emblem of the United States, Uncle Sam, is always represented as a tall, lean man, with a dyspeptic countenance. In our early history the intense struggle for existence, the fights for liberty and independence so occupied the minds of the people that they neglected their personal welfare. After the country became self-sustaining, the habit of struggling and striving, being diverted in the effort to acquire wealth, grew upon them, causing them still to neglect themselves in their desire to fill their coffers. They labored and toiled with that object in view, scarcely taking time to eat, swallowing their meals hastily without mastication, washing the foods down with liquids; taking no thought of what they were eating; only striving to get back to their employment as quickly as possible. Their close attention to work, whether physical or mental, strained to the utmost their nervous system, thus leaving an insufficient vital force to carry on digestion, assimilation and other vital processes essential to life and well being. This is one of the main causes of the prevalence of dyspepsia among the American people. Within the last two decades of the nineteenth century there has been much improvement in this particular. They are learning that, by working fewer hours in the day and taking reasonable rest, they can accomplish more than by unceasing toil and restless activity. They are beginning to learn what kind of food is best for them to eat and what are the most wholesome methods of preparing it. They are learning to spend a little more time at the table, properly masticating their food so as to get the full benefit of the product of the salivary glands; which fluid, when properly mixed with the masticated viands, constitutes the first step of digestion. They are learning much about the quality and the quantity of the food that should be taken into the stomach so as not to overload it with useless material which would interfere with the process of gastric digestion, thus rendering it unfit for the later and finer processes to be carried on in the intestinal tract. They are beginning to find out a reasonable amount of rest from labor or at least a change of occupation from physical to mental, or from mental to physical work, not only induces a better state of feeling, enabling one to get more enjoyment out of life, but also improves digestion and assimilation by resting the brain and the cerebral nerves, thus allowing the vital forces to perform their work more satisfactorily.

These and many other things our people are learning, partly from free intercourse with other nations, since travel across the ocean is so easy and inexpensive, while the financial condition of our citizens has been so greatly improved. But the principal factor in this general illumination is found in the disinterested work of the hygienic physician, who works night and day to shed light on the subjects, and who would rather see everybody in the enjoyment of good health than to enrich himself by the sickness and suffering of the community in which he lives. Yet there are many who do not hear, or hearing, do not heed the admonitions of the hygienic physician; but go on abusing the digestive apparatus by eating improper food, eating too much, taking no time for mastication, but bolting their food like a dog, and then rushing to their work, instead of taking a short period of rest to give nature an opportunity to start the work of digestion. Then, when this abuse begins to tell upon the system, and the power of nature begins to flag, they shovel in various "aids to digestion"; or if nature rebels and refuses to take what is forced upon her, they try predigested preparations or a variety of patent medicines in vain attempts to atone for their sins. Is it any wonder that they become sallow, thin, weak and miserable dyspeptics? When the stomach and alimentary canal have all their work done for them by these artificial aids, they lose the power of doing it for themselves, just as the muscles, when not exercised, loses its strength and dwindles away. But it is well known that the artificial helps are only temporary; and when nature has been weakened by their use, and when they no longer avail, the machinery halts, and the poor, dose-ridden dyspeptic pines away, lives a miserable life, dissatisfied with himself and all the world, and finally dies of inanition.

But if the persons so afflicted would give the digestive apparatus a rest, eating barely enough to keep them alive and, by the advice of their physician selecting such food as is least irritating and contains the greatest proportion of nutriment, the power of nature would assert itself, and in many instances recovery would take place without the use of medicine. The intelligent, well-informed and observant physician will see when interference becomes necessary; and instead of administering substances that do the work of digestion by proxy, will prescribe some remedy that stimulates the glandular system of the alimentary tract to secrete the necessary fluids to carry on the work of digestion.

There are many remedies that are prescribed for this purpose, some of which are very successful. I might mention one that seems to be coming to the front, although its use has not yet become universal. I refer to Seng, with which I have had some experience, and it seems to go to the fountain-head, stimulating the glands to greater secretion, thus assisting nature instead of doing her work for her. In torpid liver, which is such a common complaint and contributes so much to the making of dyspeptics, the same rule of action holds good. Some gentle hepatic stimulant like chionia would be far preferable in such cases, relieving the congestion and promoting the portal circulation without any of the bad results following the use of the powerful purgatives.

By way of recapitulation I would recommend in the treatment of indigestion, abundant rest for the alimentary system by the abstention of all superfluous food; a judicious selection of suitable nutriment, avoiding all unnecessary overloading of the stomach with indigestible substances; the absence of artificial aids to digestion, and the avoidance of predigested food, except in the case of cachetic infants; abundant rest from labor for those who have to work hard with their muscles, and plenty of moderate exercise in the open air for those who are engaged in sedentary employments.

If this course is carefully carried out, the roses will begin to bloom in the cheeks, the nerves will become strong, the muscular system will be developed, and the former weak, despondent and misanthropic dyspeptic will walk erect with a quick elastic step, strutting with health, and to use a popular phrase will be glad he is alive.

